# “It's my own fault”: Accounts and consequences of falling when living with rheumatoid arthritis

**DOI:** 10.1002/msc.1426

**Published:** 2019-08-16

**Authors:** Karly Graham, Linda Birt, Alexander MacGregor, Laura Watts, Fiona Poland

**Affiliations:** ^1^ Norwich Medical School University of East Anglia Norwich UK; ^2^ School of Health Sciences University of East Anglia Norwich UK

**Keywords:** falls, patient experiences, qualitative research, rheumatoid arthritis, risk management

## Abstract

**Introduction:**

Rheumatoid arthritis (RA) leads to biomechanical joint changes, which increases the risk of falling. The consequence of falling may be physical injury. However, the psychological consequences, including the fear of falling, can be equally important.

**Methods:**

Participants were recruited from a larger prospective study which explored the incidence of falls in people with RA. Purposive sampling considered age, sex, time since diagnosis and fall history. The recruitment site was a regional hospital. Data were collected from semi‐structured qualitative interviews and, after each fall, brief telephone interviews. Thematic analysis methods were used to investigate the psychological and social impact of falling in people with RA.

**Results:**

Twelve participants were interviewed (aged 64–85, mean 74 years; six had fallen between one and 23 times, and six had no reported falls in last 12 months). Data were supplemented with telephone notes from 287 post‐fall telephone calls. Three themes were developed: (i) the falls imaginary illustrates that the fear of falling is not dependent on experience; (ii) agentic risk management reports on the ways people self‐manage and display resilience when at risk of falling; (iii) the absence of the health professional explores the ways in which people reported being unsupported by healthcare services.

**Conclusion:**

Fear of falling when living with RA is tangible in those who have and have not fallen. This fear may limit opportunities for full participation in life. However, some people display personal resourcefulness, continuing to live purposeful lives. Understanding personal responses to falling will support the development of community interventions specific to this high‐risk group.

## 1 BACKGROUND

Rheumatoid arthritis (RA) is a systemic autoimmune disease which causes swelling, pain and biomechanical damage to joints, most usually hands, feet and knees. The disease is characterized by acute disease flares, which reduces functional ability during the flare and may lead to progressive joint damage (Markusse et al., 2015). Approximately 1% of the UK adult population face the physical and emotional challenges associated with the disease (Allen, Carville, & Mckenna, 2018). Living with RA affects quality of life (Matcham et al., 2014) and, importantly, has emotional impacts, with fears about drug treatments and longer‐term disability being common in those with the disease (Palominos et al., 2018).

RA poses a risk to independence as there is an associated increased risk of falls due to impaired mobility, balance and postural instability because of damage to lower limb joints (Armstrong, Swarbrick, Pye, & O'Neill, 2005; Stanmore et al., 2013). Falling can result in a fracture, and the risk of this is exacerbated if the person has osteoporosis, itself associated with the diagnosis of RA. (Goldring & Gravallese, 2002). Fractures after a fall contribute to increased morbidity and mortality (Deprey, Biedrzycki, & Klenz, 2017).

National strategies designed as a part of fall reduction programmes are generally generic, targeted towards older people, rather than being tailored for specific groups such as people with RA (Public Health England, 2017; ProFouND, 2019). Understanding the nature of falls specifically in people with RA is of particular importance, as these patients are a group who may already be experiencing reduced independence due to biomechanical changes.

This study addressed the research question: what is the lived experience of falling in people with RA? Understanding the reasons that people attribute to the act of falling, and their behaviour following a fall, has the potential to help to inform practice and improve the long‐term quality of life of people with RA.

## 2 METHODS

### 2.1 Research design

The research design drew on the theoretical perspective of subtle realism. In a subtle realist study, the epistemological position is that social phenomena exist independently of the person; however, the understanding of phenomena is only known through the individual's representation of them (Duncan & Nicol, 2004). So, in RA, the increased risk of falling is an objective fact, but the person's knowledge and understanding of falling is grounded within their experiences; their knowledge is socially constructed (Gray, 2013).

### 2.2 Recruitment and sample

Participants to the present interview study were recruited from another study, exploring the incidence of falls in people diagnosed with RA at a regional hospital. Inclusion criteria were people aged over 60 years, who had a clinical diagnosis of RA made by a rheumatologist and were attending an outpatient RA clinic. Participants returned a monthly record sheet on which they recorded falls for a 12‐month period. If they had fallen, they were contacted by telephone to take part in a short interview. The sample for face‐to‐face interview were purposively selected from a cohort of 30 people who reported falling and 30 people who did not report falling within the large study. We sampled for age, sex, time since diagnosis and number of falls.

### 2.3 Data collection

Data were collected by face‐to‐face semi‐structured interviews undertaken by the first author. The majority of interviews took place in participants' homes, with two people choosing to be interviewed in a private room at the hospital outpatient department. A topic guide was developed after reviewing the literature on experiencing falls and the effects of falls, and drew on the clinical experience of the research team. The questions were designed to elicit accounts about the diversity of experiences of living with RA, including support from statutory services and any adaptations people themselves had made in the light of their views, values and practices. People were asked about their mobility and if they had experienced falls. Follow‐up questions were designed to enable a detailed understanding of participants' own views on the reasons for their fall, and the physical and emotional impact of falling. This approach enabled us to contextualize experiences, so gaining insights into how participants engaged with their social world, how this was affected by personal experiences of living with RA and how falls might be understood in this social context (Parahoo, 2006). Interviews lasted 15–65 min; all face‐to‐face interviews lasted over 30 min. Recordings were made, and data were transcribed, anonymized and entered into a database (Table 1).

**Table 1:** Interview topic guide

**1**	**Can you tell me something about your life before you knew you had RA?**
	Prompts: Age? Work? Family?
**2**	**What happened when you were diagnosed with RA? What changed?**
**3**	**After diagnosis, was anything done to help you to manage things for yourself?**
	Prompts: Who helped? HCPs? Any therapy? Progress in early days?
**4**	**How do you manage your mobility, living with your RA?**
	Prompts: Concerns about falls? Experience of falls?
**5**	**What would help with your mobility? What did you hope for/expect?**
**6**	**What if any special home or family arrangements do you have which help you get on with life with RA?**
	Prompts: Paid/unpaid support?
**7**	**What worries, if any, do you have about falling?**

HCP: healthcare professional; RA: rheumatoid arthritis.

Interview data were supplemented by post‐fall telephone interviews. Here, participants were asked about the cause of the fall, any injury and the treatment, including hospitalization. They were also asked if their mobility inside and outside the home had become more difficult.

To gain a more detailed understanding of the experience of falling and any fear of falling, we also carried out a face‐to‐face interview study with a subset of people from the main study. Information already obtained on sex, age, length of time with RA and number of falls enabled purposive sampling to take place, for the interview study. Recordings were made during these interviews, and were transcribed, anonymized and entered into a database.

### 2.4 Data analysis

Data analysis drew on the principles of thematic analysis outlined by Braun and Clarke (2006). The process was iterative: after initial familiarization and agreement on the coding framework, data were coded by the first author. The team then reviewed codes and developed initial themes. Initially, we named nine inductive themes which covered the impact of diagnosis, physical effect of RA, fear of falling, types of support and treatment. These themes were again reviewed by the whole research team, which included clinical specialists and qualitative researchers, and two Patient and Public Involvement (PPI) members; at this stage, we defined three interpretative themes. The credibility of the results was enhanced by repeated team meetings, which enabled professionals from different academic and clinical backgrounds to challenge and extend interpretations. The patient perspective was supported by including PPI colleagues. The second author joined the study partway through, and carried out a review of the analysis; no new codes or themes were identified.

### 2.5 Ethical approval

Ethical approval for the study was obtained from National Research Ethics Service East of England committee (11/EE/0335) and written informed consent was obtained from all participants. Data were collected between May 2013 and July 2014 by the first and fourth authors.

## 3 RESULTS

The main study (https://www.fundingawards.nihr.ac.uk/award/PB-PG-0808-14201) recruited 437 participants, of whom 81 reported falling during the 12‐month period. Participants experienced between one and 23 falls. Participants were contacted after each fall, and 287 telephone interviews were undertaken. Only 12 falls resulted in an identified fracture. Approximately half the people (*N* = 158) reported that, after the fall, they had experienced difficulty in mobilizing in and outside the home.

Following purposive sampling from the main study data set, 12 participants took part in face‐to‐face interviews. Six had reported falling [“faller” group], and six had not reported falls [“non‐faller” group]. The sample was matched for gender across the two groups, with four female and two male participants in each group, RA being two to three times more common in females. There was a variation in age, ranging from 64 years to 85 years, with a mean of 74 years. Time since diagnosis ranged from 6 years to 30 years. In the faller group, the number of falls ranged from one to 23 in the past 12 months (see Table 2).

**Table 2:** Interview study participant characteristics

Patient ID	Sex	Age	Years since diagnosis	No. of falls over 1 yr	Patient ID	Sex	Age	Years since diagnosis
F422	F	70	26	11	NF642	F	71	18
F060	M	85	30	1	NF068	M	84	18
F134	F	76	10	3	NF200	F	71	6
F501	M	78	6	17	NF127	M	77	18
F154	F	75	10	23	NF268	F	66	25
F285	F	64	24	2	NF473	F	64	12

F: female; ID, identification; M: male.

### 3.1 Themes

All participants gave accounts of trying to live actively with RA, but in each group there were examples of how the disease process restricted their activities. In talking about falling and fear of falling, they offered complex accounts which detailed the ways in which they assimilated falling into the practicalities of their everyday living. Participants did not see falling simply as a symptom of RA, but as having important and notable consequences. We now present a detailed analysis under three themes: “*the falls imaginary*,” envisaging the causes and consequences of falling; “*agentic risk management*,” revealing the ways that participants themselves mitigated risk; and “*the absence of the health professional*,” reporting where and how health professionals had been present in the participants' care, yet absent within accounts of falling.

#### 3.1.1 The falls imaginary

The accounts provided in response to questions about “fear of falling” indicate that thoughts and emotional responses to the risk of falling go beyond mere emotional responsive fears but show how the participants actively constituted a cultural “falls imaginary,” combining ideas, values and interactive interpretations to anticipate and respond to the prospect and experiences of falls. The imaginary of the “fall” is therefore contextualized within their accounts, presenting causes and consequences of the event.

##### The cause

A fall could be imagined as an occasion when one physically landed on the ground, and participants clearly made a distinction between falling and a trip:

I have tripped hundreds of times but there has always been something to grab onto indoors. (642 F 71, non‐faller)

When someone trips, it appears that lucky chance can be the factor which prevents a fall to the ground.

Biomechanical changes inherent in RA appeared to be the “objective” cause of many falls, although participants themselves described this in nonmedical language; explaining that knees and ankles “give way” (*“*knees gave way and I went down*”*; telephone interview). Participants also described the cause of falls as “disobedient feet”:

Because you're not picking your feet up much, are you? You're sort of shuffling along rather than walking properly. (285 F 64, faller)

They (feet) don't move quick enough, that's the trouble to save yourself … they're cold so they're stiff and they won't move quick enough. (501 M 78, faller)

Many participants said they were dizzy and unbalanced before a fall: “dizzy getting out of bed”; “cleaning the kitchen, bent down and lost my balance” (telephone interviews). One felt that an RA flare increased risk: “I'm more likely to fall when I'm unwell [RA flare].”

Rather than a biomechanical cause, several participants attributed a fall to “not concentrating” or falling over an object:

I fell over the box and I smashed the birdcage. But that was my own fault, I wasn't looking. I was trying to do three jobs at once. (134 F 76, faller)

I just walked out there, turned round, tripped over something someone that had left in the way, because all the shoes get left out there of course. And I went smack, smack down. (422 F 70, faller)

Occasionally, accounts of reduced concentration, or falling when tired, were accompanied by a comment about the need to pace oneself. Blaming their own physical ineptitude or lack of concentration enabled people to retain agency and therefore the potential to continue to control their functioning themselves. No one reported having received any recent advice on pacing or reducing environmental risk in order to help them take steps to reduce risk of falling.

Some participants presented the fall as occurring as part of everyday living, and so apparently unconnected with RA, preserving a longer‐term continuity of everyday living. For example, one person said that they had been hit by another skier on holiday. Others explained that the environment had been the cause of the fall, such as uneven ground and equipment. For example, in the telephone interviews, one described sitting on a stool on uneven ground and falling off, and another sitting on a golf trolley which rolled away. Another telephone interviewee explained that he had tripped over the base of the ladder while up on a flat roof. The range of activities and environments in which falls occurred suggests that people were not always curtailing activity because of their RA, and that the falls imaginary was not routinely given greater credence of risk simply because of having an RA diagnosis. Therefore, it may be important not unquestioningly to attribute falls with impairment related to RA.

##### The consequence

The fear of falling was present in the imaginary of those who had never fallen, and here concern was situated in the way that one would appear to others:

If I fell, I would have to go on all fours and then get up. I'd die! Totally embarrassing. (200 F 71, non‐faller)

Even a change in gait, an indicator of impairment and a frequent precipitator of falling, could be something that needed to be hidden:

I'm becoming—it sounds awful saying it—but I am more unsteady because I don't think anyone else notices. (207 F 71, non‐faller)

One participant explained that they did not use a stick as this would make them feel and appear old.

It did not appear to be the frequency and number of falls which increased a participant's concern about falling. Rather, it was the potential or actual emotional consequences which seemed to shape the imaginary of falling. Some people focused on falling and had heightened concern:

… began to worry that if I weren't thinking about what I was doing, I was going to fall and that gradually got me more down and down. (285 F 64, faller)

However, the most frequent faller explained:

You just don't take no notice of it—just used to it. It was only when we were doing the Falls diary—then it hits you. I've fallen there [just outside house] quite a bit. (154 F 75, faller)

Yet, there is contradiction in this participant's account, as she also stated that she was fearful of falling when out, and lacked confidence. For others, fear of falling was considered in relation to other priorities in their lives. One participant fell frequently but the possibility of cancer returning was of greater concern for harm to themselves:

If I fall over, I fall over. Cancer that's the thing I worry about coming back more than anything really. (134 F 76, faller)

Others had had falls years ago, unrelated to RA, and the consequence of this now had an impact on their activity:

I had a nasty bike accident … and its left me with short‐term memory problems … now I am absolutely paranoid about falling over. (473 F 63, non‐faller).

#### 3.1.2 Agentic risk management

The priority given by participants to their agency in their falls imaginary helped to explain the attention they paid to their own agentic role in managing their own risks. Participants who had fallen and those who had not fallen could be seen to situate their accounts within their articulated descriptions of the steps they had devised and taken to reduce the risk of falls. Those who had experienced falls drew on this to inform their construction of such steps:

Since I had a fall, I take a stick with me all the while. I can't go out the house without it. When I tripped on the back door step, I was coming in and I don't know what I done. I got a stick in my hand and I just went down … that was the one that made me realize I needed the stick with me all the while, when I am out and about. (285 F 64, faller).

However, daily activities meant that the stick was not always used (for example: “I'm alright in the garden … well, you can't hang washing out if you've got a stick”)*,* suggesting that the risks of falling were being weighed up alongside the need to perform activities.

Non‐fallers also reported self‐management strategies to reduce risk of falling:

You use common sense … you do become, not risk adverse … but you do a safety check—you think, I will get the stepladder round rather than trying to reach. (127 M 77, non‐faller)

Many had made what might be seen as positive adaptations: they had had grab rails installed, they used walking aids and they used showers, rather than the bath. Some had “tinkered” with equipment, to make it useable for themselves, such as adapting the garden bin so that they could open it with one hand while holding a stick. In a telephone interview, a participant explained that they had rearranged the garden furniture so that if they were to fall, they could use it to get up independently. These were all examples of how people had, in agentic ways, made changes to increase independence and reduce the risk of falling and negative consequences of falling.

However, other types of agentic action might have detrimental impacts on health and well‐being, as some participants actively chose to withdraw from a range of activities:

I'm fearing to fall, so I just don't go out. I don't like [going out]. In fact, the only thing I'll do is go into a supermarket with [carer]. It's confidence—I lack the confidence now. I won't get out of the chair now … because I feel all sort of whooo. (154 F 75, faller)

The gardening … I used to like being outside, doing things outside. But now I'm scared I'm going to fall over every time I go out there. (285 F 64, faller)

Even those with no history of a fall in the past 12 months gave accounts of modifying behaviour to reduce a perceived risk:

A friend and I had lunch; we went to restaurant and coming out, those steps—well, I didn't notice it and, anyway, we were talking and you don't think about it. And she was ahead of me and I said: “Oooooh—I don't like this!” So, we just laughed it off. I won't go there again … if there's a slope, I'll use a slope. (200 F 71, non‐faller)

Telephone interview data showed that many people experienced increased difficulty with mobilizing in and outside the home after a fall.

#### 3.1.3 The absence of the health professional

There were 287 post‐fall telephone interviews, yet only a minority of people reported receiving medical care. Eleven people were taken to hospital by ambulance; eight people self‐reported attending accident and emergency units. From these 19 people, six had hospital stays of between 5 days and 13 days. A further 15 people were seen in primary care, with no resulting hospital stay. These data indicate that the majority of people do not have contact with medical professionals after a fall, instead managing the physical and psychological impacts themselves, with varying success.

The absence of the health professional at the time of the fall is consistent with accounts of access to health professionals during the course of the disease, suggesting that this cohort do not expect, or seek, professional support. The lack of help‐seeking may be contextualized within previous negative experiences. Several participants spoke about their contact with various health professionals; this was often reported as inadequate in terms of engaging with and supporting the person's own priorities and activities:

I only went twice. I went [second time] after a fortnight and she said “Carry on with the exercises.”' I said, what's the sense in going and not getting anywhere? (134 F 76, faller)

This led to people self‐managing not only daily activities, but also medication management. One participant described the conflict between treatment aims, as in one clinic she was told to lose weight, which she felt she could not do while on steroids, yet in the RA clinic there was reluctance to take her off steroids. The outcome was:

So I took them off myself … I'll do it my way and I did it, slowly. (134 F 75, faller)

There appeared to be an absence of communication between professionals, and participants were left wondering what was going on and what they could do to manage their disease:

There's no follow‐through when you see your GP. There doesn't seem to be a link between GP/consultant, accessed atconsultant/GP. You're somewhere in the middle—saying, what's going on. (473 F 63, non‐faller)

Others, however, did find the advice of health professionals invaluable, although it was not reported here in reference to falls management or risk reductions. Advice included exercises and how to access grants for home modification, and one participant explained that they could self‐refer back into a service:

They're [orthotics] really brilliant, as I only have to ring and he'll see me again (285 F 64, faller)

When participants had goals that they believed would improve their quality of life, they pursued these in the absence of health professional advice:

I couldn't do the decorating until I started some different exercises; I found them for myself, and then I could get my arms up for a little while, just to do around the edges. (501 M 78, faller)

None of the participants, in either the telephone or face‐to‐face interviews, reported being asked about falls by health professionals, or being referred either to the secondary care dedicated falls clinic or the community falls service, at the time of their fall, yet this is a population with potentially complex medical care needs.

## 4 DISCUSSION

The present study found that people living with RA have some fear of falling, whether or not they had a history of falls. Such fear was situated within accounts of previous falls and, importantly, demonstrated an awareness of biomechanical change in the lower limbs. We have defined the falls imaginary as a cultural, social construct, where people combine experiences, values and expectations to anticipate the prospect, and respond to experiences of falling. This enables a developed understanding of the complex concerns of people with RA about falling. Participants situated concerns in relation to injury risks and loss of independence; they set these alongside the emotional consequences of embarrassment and damaged presentation of self to others. People demonstrated their agency in minimizing falls risks. However, this could be seen sometimes to lead to their selecting to reduce social participation and reducing previously enjoyed activities, such as gardening. They also took actions to avoid the embarrassment of falling; for example, they described directing their attention to their use of outdoor space. These agentic risk management initiatives appeared, notably, to be undertaken without recourse to healthcare professional interventions.

People with RA have a greater fear of falling than those without RA (Akyol et al., 2018). This fear may be justified, as biomechanical and physiological disease processes affect balance, and increase fatigue and subsequent risks of falling (Brenton‐Rule, Dalbeth, Bassett, Menz, & Rome, 2015; Brenton‐Rule, Dalbeth, Menz, Bassett, & Rome, 2016). Loss of balance was frequently reported by our participants. However, they located their loss of balance within the activity they were undertaking, highlighting the relationship between the disease process and everyday life.

Within our study, people rarely had contact with healthcare professionals, rather presenting themselves as experts in their care by managing their own education and actively making adaptations to activities and equipment. This resonates with the results of Erwin, Edwards, Woolf, Whitcombe, and Kilty (2018), who found that people with arthritis wanted community healthcare professionals to recognize the expertise of many people in managing their disease. Nonetheless, they also noted that people wanted community healthcare professionals to offer advice on pacing and pain management. Our study highlights a dearth of healthcare advice pre‐and post‐falls, which appears to conflict with advice on best health and social care practice (National Institute for Health and Care Excellence, 2013; Stanmore, 2015). This aspect of practice therefore requires further investigation, to determine its potential to reduce the risk of repeated falls, and any consequent injury or loss of independence.

We found that the number and frequency of falls seemed to be surprisingly incidental to a person's attitude and concern about falling. It may be that fear of falling becomes superseded by more pressing, recurring everyday concerns, such as the management of pain and fatigue, or fear of treatment failure. Certainly, the treatment trajectory in RA can be characterized by unsuccessful drug treatment or side effects from treatment regimens (McArthur, Birt, & Goodacre, 2015; Palominos et al., 2018). However, many people in our study demonstrated resilience in their ability to make changes to their activities, homes and aids, without recourse to health professionals. Although for many people such changes seemed to work, in some key examples such changes reduced social participation. It is here that timely multidisciplinary intervention would enable people to maintain activity and quality of life (Stanmore, 2015).

### 4.1 Strengths and limitations

Using interviews enabled participants to talk in specific detail about their falls within the context of their wider life and their stage of disease. Nonetheless, this sample was relatively restricted, and drawn from one regional population and service. Future work might explore whether other healthcare services have evolved different support pathways, with different outcomes, from enhanced services for falls in RA. Within this qualitative work, we did not collect data specifically on comorbidity, although such information may provide greater context to such results. Relevant here is the finding of Jamison, Neuberger, and Miller (2003) that people with RA expressing most fear of falling had more comorbid conditions than those who denied a fear of falling. It may be that the physical or cognitive consequence of other diseases affects falls behaviours in this population.

## 5 CONCLUSION

Fear of falling in people with RA is a complex concept, shaped not only by biomechanical changes which increased risk of falling, but also by personal and social factors. Although fear of falling can lead to reduced involvement in daily activities, for many people their personal resourcefulness meant that they actively adapted daily routines so as to manage the risk of falling in ways that they themselves found acceptable. Interestingly, there was little reported evidence of support from health professionals in managing the fear of falling or consequences of a fall. A deeper understanding of how people with RA manage falls could help in developing community interventions specific and acceptable to this high‐risk group of people.

## CONFLICTS OF INTEREST

The authors declare no conflicts of interests.

## AUTHORS' CONTRIBUTIONS

F.P. and A.M. made substantial contribution to the conception and design of the study. R.G., L.W. and L.B. made substantial contributions to the collection, analysis and interpretation of the data. The manuscript was drafted by K.G. and L.B., and all authors then made comments and amendments. All authors agreed to the submission of the manuscript.
